# Assessing the Experience and Management of Acute Post-Operative Pain from Caesarean Delivery: A Multi-Centre Cohort Study

**DOI:** 10.3390/jcm14134638

**Published:** 2025-06-30

**Authors:** Carles Espinós Ramírez, Gisela Roca Amatria, Pere Castellví Obiols, David Martínez-Rodríguez, Mireia Raynard, Blanca Viscasillas Draper, Paula Masgoret, Cristina Rodríguez Cosmen, Laura Subirana Giménez, Maria Martinez García, Gerard Mestres, Martha Melo, Alèxia Nebot Galindo, Natàlia Montero Gaig, Virginia Sánchez-Migallón, David Valencia Royo, Nuria Lara Pacheco Comino, Inés Bermejo Perez, Cristina Santos Farré, Lluís Toll Salillas, Arnau Alonso Gelabert, Marta Homs, Patricia Ribas, Claudia Teixell, Ana María Plaza Moral, Bea Tena, Adrián Fernández Castiñeira, Mireia Armengol Gay, Beatriz Fort Pelai, Carolina García Bartoló, Carolina Mestre Iniesta, Anna Peig Font, Paula Gil Esteller, Jean Louis Clave, Sandra Gasca Pera, Astrid Batalla, Verónica Vargas Raidi

**Affiliations:** 1 Hospital Universitari Germans Trias i Pujol, 08916 Badalona, Spain; bviscasillasdr.germanstrias@gencat.cat (B.V.D.); d.valenciaroyo@gmail.com (D.V.R.); nuriapacheco97@gmail.com (N.L.P.C.); bermejoperezines@gmail.com (I.B.P.); cristina.santosfarre@gmail.com (C.S.F.); lltollsalillas@gmail.com (L.T.S.); arnaualonsog@gmail.com (A.A.G.); 2 Department of Medicine, School of Medicine and Health Sciences, Universitat Internacional de Catalunya (UIC Barcelona), 08017 Sant Cugat del Vallès, Spain; pcastellvi@uic.es (P.C.O.); mariamartinezgarcia@gmail.com (M.M.G.); annapeig@hotmail.com (A.P.F.); paula.gil.esteller@gmail.com (P.G.E.); 3 Hospital Universitari Sagrat Cor, 08029 Barcelona, Spain; giselaroca@gmail.com; 4 Instituto Universitario de Matemática Multidisciplinar, Universitat Politècnica de València, 46022 Valencia, Spain; vidda.martinez@gmail.com; 5 Hospital Universitari Dexeus, 08028 Barcelona, Spain; mireiaraynard@hotmail.com (M.R.); martahoms7@gmail.com (M.H.); pribascarrasco@gmail.com (P.R.); claudiateixell@gmail.com (C.T.); 6 Hospital Clínic de Barcelona, 08036 Barcelona, Spain; pmasgoret@gmail.com (P.M.); aplaza@clinic.cat (A.M.P.M.); btena@clinic.cat (B.T.); 7 Hospital del Mar, 08003 Barcelona, Spain; crodriguez@psmar.cat (C.R.C.); afernandezcastineira@psmar.cat (A.F.C.); marmengolgay@psmar.cat (M.A.G.); bfort@psmar.cat (B.F.P.); 8 Consorci Sanitari Parc Taulí, 08208 Sabadell, Spain; lsubiranagimenez@gmail.com (L.S.G.); carolinagarciabartolo@gmail.com (C.G.B.); carolina.mestre.iniesta@gmail.com (C.M.I.); 9 Consorci Sanitari de Terrassa, 08227 Terrassa, Spain; 10 Hospital de la Santa Creu i Sant Pau, 08041 Barcelona, Spain; gmestresg@gmail.com (G.M.); mmelo@santpau.cat (M.M.); abatalla@santpau.cat (A.B.); 11 Consorci Sanitari del Maresme, 08304 Mataró, Spain; anebot@csdm.cat (A.N.G.); vvargas@csdm.cat (V.V.R.); 12 Consorci Sanitari Integral, 08906 Esplugues de Llobregat, Spain; natalia.montero@gmail.com; 13 Hospital Universitari Vall d’Hebron, 08035 Barcelona, Spain; virginia.smp@gmail.com; 14 Hospital Sant Joan de Déu, 08950 Barcelona, Spain; 15 Hospital Universitari Mútua de Terrassa, 08223 Terrassa, Spain; jeanluclave@gmail.com (J.L.C.); sgascapera@gmail.com (S.G.P.)

**Keywords:** caesarean section, pain, postoperative, pain measurement, analgesia, obstetric

## Abstract

**Background:** Caesarean section is considered one of the surgeries with the highest prevalence of postoperative pain, yet this is often underestimated and undertreated. This study was aimed at evaluating the prevalence and severity of postoperative pain, assessing which analgesic strategy is the most effective and identifying those risk factors associated with poorer analgesic results. **Methods:** A multi-centre observational study was conducted on 514 women undergoing elective caesarean section. The primary endpoints included postoperative pain severity at rest and with movement at 6 and 24 h. **Results:** The combination of intrathecal morphine and fentanyl with acetaminophen and Non Steroid Anti-inflammatory Drugs (NSAIDs) was associated with better pain control than any of the following treatments: intrathecal fentanyl with systemic acetaminophen and NSAIDs (2.49 ± 2.04 vs. 3.91 ± 2.75, ES = −0.610, *p* = 0.01), elastomeric pump at 6 h at rest (2.49 ± 2.04 vs. 4.10 ± 2.86, ES −0.733, *p* = 0.04) and with movement (4.44 ± 2.41 vs. 6.14 ± 3.08, ES −0.671, *p* = 0.01) or epidural analgesia (4.44 ± 2.41 vs. 5.65 ± 2.57, ES −0.496, *p* = 0.02). No risk factors predicting poorer postoperative analgesia were found. **Conclusions:** The prevalence of postoperative pain control after elective caesarean section is high. The best analgesic postoperative regimen includes intrathecal morphine together with fentanyl and systemic analgesics. No risk factors associated with poorer outcomes were found.

## 1. Introduction

Delivery by caesarean section is one of the most common surgeries performed in the world, with increasing global rates [1,2]. It is associated with a prevalence of postoperative pain [3] as high as 60% according to recent studies [4]. Yet, this pain is often underestimated, underrecognized and poorly treated in clinical practice, thus generating a gap between the expected and the actual standard of care for this population.

One of the factors contributing to the poor analgesic management of postpartum pain is a cognitive concern about the potential impact of analgesic drugs on the newborn’s health [5]. This fear frequently leads to mothers not reporting their severe pain. While it is true that some systemic analgesics may pass into breast milk and cause adverse effects on the newborn [6], there is lack of evidence of similar complications when using other alternatives. This misconception or uncertainty may lead to the unnecessary withholding of appropriate analgesic therapy. Poorly-managed postoperative pain can lead to a wide range of adverse consequences such as delays in women’s recovery, increased opioid consumption, impaired mother-child bonding caused by a limitation in breastfeeding [7], and a negative impact on physiological and psychological maternal wellbeing [8]. Additionally, severe postoperative pain is a well-documented risk factor for the development of chronic postsurgical pain [9].

Several analgesic approaches have been explored with the aim of improving post-surgical pain management after caesarean section, such as adding adjuvants using neuraxial techniques (e.g., dexmedetomidine [10]) or alternative local and regional blockages such as quadratus lumborum or transversus abdominis plane blocks [11]). Additionally, non-pharmacological techniques [12] have been investigated as part of a multimodal analgesia regimen. Although some promising analgesics have appeared during the last few years, most of them have not demonstrated a clinically significant value in improving acute pain [13], therefore they have not been widely adopted as standard practice. A rigorous comparison of different strategies is needed to better find the best method to manage postoperative pain after elective caesarean section. In addition, preoperative reidentification of those patients with an increased risk of presenting with moderate or severe postoperative pain could potentially lead to implementing patient-based individualized complex strategies [14].

This study was aimed at achieving the following goals: (1) evaluating the prevalence and severity of acute postoperative pain after caesarean section, (2) comparing the pain outcomes of various intraoperative and postoperative analgesic strategies and (3) identifying those factors associated with increased postoperative pain syndromes.

## 2. Materials and Methods

### 2.1. Study Design

A multi-centre prospective cohort study with 24 h of follow up was conducted in 10 Spanish hospitals between 15 March 2023 and 24 April 2024. The follow up ended on 25 April 2024. This research obtained approval from the ethics and research committee of the Consorci Sanitari de Terrassa on 6 February 2023, (02-23-270-003) and was registered at ClinicalTrials.gov (NCT05739747) on 13 February 2023. The lead investigators at each centre also received approval from their respective ethical research committees. This research followed the Strengthening the Reporting of Observational Studies in Epidemiology (STROBE) Statement [15].

### 2.2. Participants

All women scheduled for elective caesarean section under subarachnoidal anaesthesia were informed about this research and invited to participate prior to the surgical procedure. Those presenting with the following qualities were excluded: (i) underaged women (<18 years old), (ii) patients diagnosed with an unstable psychiatric or psychological disorder, (iii) women presenting with a language barrier or limited communication challenges, (iv) women not undergoing the classical surgical and anaesthetic approach (pfannestiel incision and subarachnoidal anaesthesia) and (v) women refusing to give consent to participate in the research.

### 2.3. Outcomes

The primary endpoint was pain severity after surgery. A secondary analysis was performed according to the type of subarachnoidal anaesthesia and postoperative systemic analgesia utilized.

### 2.4. Data Collection

The coordinating investigators of each hospital’s local research team underwent training sessions on how and when to complete all the questionnaires prior to the start of the recruitment.

Prior to surgery, the following data were collected from the patients’ interviews and medical records: (1) relevant medical and gynaecological history; (2) smoking and alcohol consumption and history of substance use disorders; (3) physical status as per the America Society of Anaesthesiologists’ classification; (4) presence of pain over the surgical area and in other body parts and (5) need for chronic analgesic prescription. Additionally, the following questionnaires were administered: the Spanish version of the Pain Catastrophizing Scale (PCS) [16] and the second Spanish version of the Short Form Health Survey-12 (SF-12) questionnaire [17].

Pain severity at rest and at movement was measured at 6 h and 24 h after surgery. Pain severity was assessed with the verbal numerical rating scale (VNRS) from 0 to 10 (0 being no pain and 10 the worst imaginable pain). Values were classified categorically into no pain (NVRS = 0/10), mild pain (between 1 and 3), moderate pain (between 4 and 6) and severe pain when ≥7/10.

Data regarding the anaesthetic process was also obtained, including the following: (1) type and doses of adjuvant intrathecal drugs administered and (2) type of systemic analgesic administered.

All clinical data were collected and stored in an anonymized database hosted on a web server that was accessible from a web application. The platform complied with the General Data Protection Regulation. The data was stored on European territory subject to the confidentiality and ownership established by law.

### 2.5. Sample Size

The results presented in this paper are from a sub-analysis of a study with the objective of validating the Gendolcat presurgical model for chronic postsurgical pain [18] after caesarean section. The sample calculation was made assuming a 20% prevalence of pain [19]. Using a desired level of confidence of 95%, a margin of error of ±0.04 and a 30% loss to follow-up, a minimum of 499 participants was needed.

### 2.6. Comparison of the Different Analgesic Techniques

One of the objectives of the study was to assess which analgesic techniques may be the best to decrease postoperative pain. Among all the techniques, three have stood out: (i) intrathecal morphine (M), (ii) intrathecal fentanyl (F) and (iii) the combination of intrathecal morphine and fentanyl (M + F). Moreover, the analgesia provided during the surgery was combined with the different analgesic strategies after these strategies were tested. Five groups were analysed: (i) intrathecal fentanyl with a combination of acetaminophen and nonsteroidal anti-inflammatory drugs (NSAIDs) (IF), (ii) intrathecal fentanyl with an elastomeric pump (understood as a constant intravenous perfusion of 200 mg of dexketoprofen and 400 mg of tramadol for 48 h) of anti-inflammatory and weak opioid drugs (IF + EL), (iii) intrathecal fentanyl and epidural analgesia with 8 mL/h of ropivacaine 0.05% and 2 mcg/mL of fentanyl for 48 h (IF + EP), (iv) intrathecal morphine with acetaminophen and NSAIDs (IM) and (v) the combination of intrathecal morphine and fentanyl with acetaminophen and NSAIDs (IF + IM). Finally, the differences between the different dosses of morphine provided during the anaesthesia were tested. The main goal was to find out if there was any difference between the doses of >100 mcg and the doses of ≤100 mcg. Also, the postoperative pain between the doses of >1 mcg/kg and the doses of ≤1 mcg/kg was recorded. The rationale for establishing this lower threshold is based on the observed correlation between cerebrospinal fluid (CSF) volume and body mass index (BMI). Additionally, the definition of low-dose intrathecal morphine varies considerably in the literature, typically ranging from 50 to 100 µg, further supporting the need for weight-based dosing to ensure consistent drug distribution and therapeutic effect.

### 2.7. Statistical Analysis

Quantitative data is expressed as mean and standard deviation (SD). Qualitative variables are expressed as frequencies and percentages. The normality of the samples was tested with the Shaphiro–Wilk test. The equal variance of two samples was calculated with the Levene statistical test. The correlation between two variables was measured with Pearson’s Correlation for variables with a normal distribution, and by Spearman’s Rank Correlation otherwise. Linear regression models were developed to find a relationship between the independent variables and postoperative pain. Effect size (ES) was calculated with Cohen’s d if normality and homoscedasticity were accomplished, otherwise it was calculated with Glass’ d. ES were classified as small from ≥0.2 to <0.5; medium from ≥0.5 to <0.8 and large when ≥0.8. Mean differences between two groups were tested with the Student *t* test if normality and homoscedasticity for the groups were accomplished, otherwise, the Mann–Whitney U test was used. The distribution difference of a quantitative variable between multiple groups, where normality, homoscedasticity or similar sample size was not accomplished, was tested with Dunn’s Post-hoc Test with a Bonferroni *p* value adjustment. All intervals were calculated with a 95% confidence level and tests were considered significant for *p*-values below 0.05. All analyses were performed with the Python3 programming language, pandas 2.2., numpy 1.26.0, scipy 1.14.1 and scikit-learn 1.5.2.

## 3. Results

Data from 10 different hospitals were collected and analysed, with a total of 514 women. Six cases with relevant missing or anomalous data were excluded.

A descriptive analysis of the main variables of the population sample studied is shown in Table 1. There was no correlation between preoperative SF-12 or PCS total scores and the severity of postoperative pain. There was a significant association between alcohol consumption and pain scores after 6 h at rest (Student’s *t* test = −2.175, *p* = 0.03). No correlation between any type of postoperative pain and the existence of obstetric risk or previous caesarean was found. Several linear regression models were built to identify which variables were independently associated with increased postoperative pain scores, but none yielded statistically significant conclusions.

**Figure 1:**
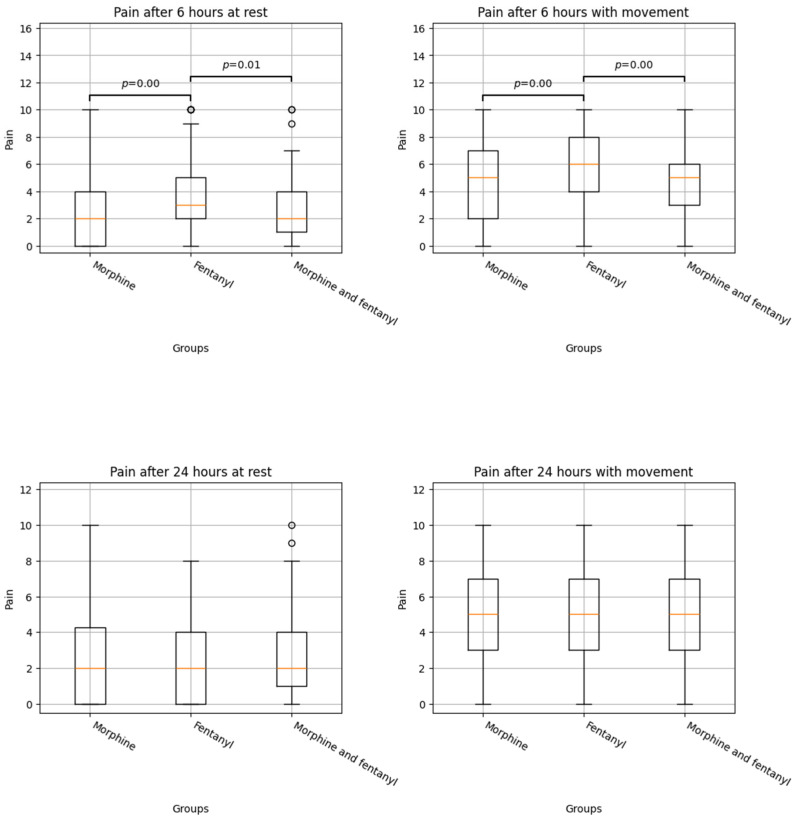
Boxplots of pain distribution by anaesthesia technique. Statistical differences between the distributions and the *p* value of the test are shown above the boxplots.

There was better pain control in the M group compared to the fentanyl F group at 6 h at rest (2.44 (2.60) vs. 3.59 (2.7), ES = 0.435, *p* < 0.001) and with movement (4.66 (2.97) vs. 5.71 (2.71), ES = 0.370, *p* = 0.004). We found similar results comparing the morphine combined with fentanyl (M + F) group to the F group at 6 h at rest (2.65 (2.24) vs. 3.59 (2.70), ES = 0.088, *p* = 0.012) and movement (4.78 (2.48) vs. 5.71 (2.71), ES = 0.044, *p* = 0.004). No differences were observed between the different groups at 24 h either at rest or with movement. Postoperative outcomes are shown in Table 2 and Figure 1.

**Figure 2:**
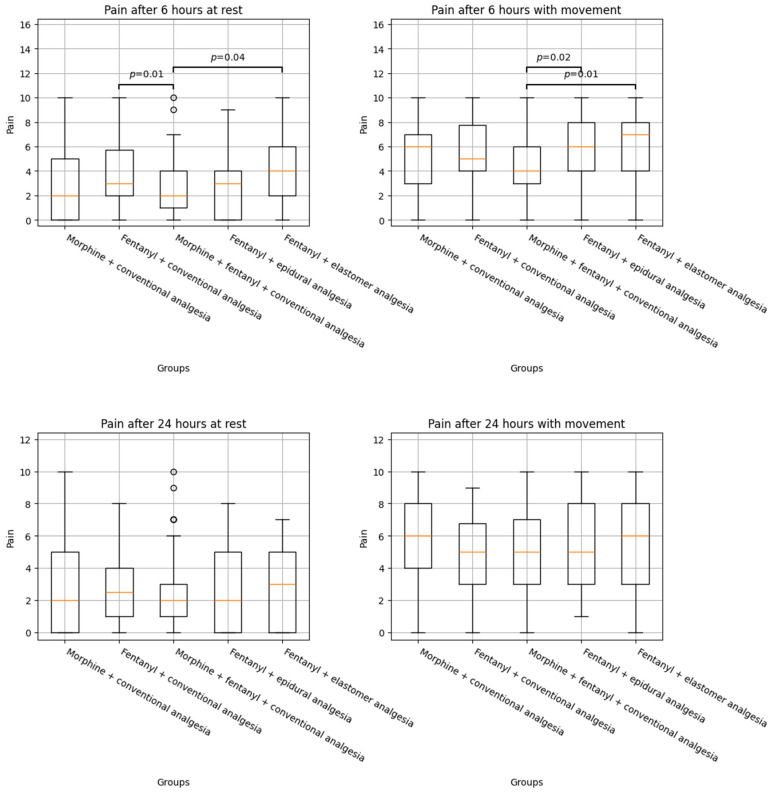
Histogram of pain distribution by anaesthesia and analgesia dose. Statistical differences between the distributions and the *p* value of the test are shown above the boxplots.

Table 3 shows the results of the comparison between the different intraoperative and postoperative analgesic combinations. Better pain control was observed in the IF + IM group compared to the IF (2.49 (2.04) vs. 3.91 (2.75), ES = −0.610, *p* = 0.01) or IF + EL groups (2.49 (2.04) vs. 4.10 (2.86), ES = −0.733, *p* = 0.04) at 6 h at rest. This analgesic strategy was also superior compared to IF + EL (4.44 (2.41) vs. 6.14 (3.08) and ES = −0.671, *p* = 0.01) and IF + EP analgesia at 6 h with movement (4.44 (2.41) vs. 5.65 (2.57), ES = −0.496, *p* = 0.02). Although a similar trend was observed when comparing IF (4.44 (2.41) vs. 5.49 (2.62), ES = −0.311, *p* = 0.09) and IM (4.44 (2.41) vs. 5.25 (2.79), ES = −0.421, *p* = 0.06) at 6 h with movement, no statistically significant differences were observed. No differences were observed either at rest or with movement 24 h after surgery. The boxplots of the variables’ distributions can be observed in Figure 2.

The comparison of pain ratings at 6 or 24 h at rest or with movement yielded no differences between those women receiving >100 mcg or ≤100 mcg intrathecal adjuvant morphine. Postoperative pain at rest at 6 h was significantly lower in those participants receiving >1 mcg/kg intrathecal adjuvant morphine (2.27 (2.70) vs. 3.00 (2.21), ES = 0.287, *p* = 0.023), but the difference was not significantly maintained at 6 h on the move or at 24 h. The results are shown in Table 4.

## 4. Discussion

The prevalence of moderate or severe pain after caesarean section surgery in this study is similar to the results of already published studies [20]. Although it has been considered one of the surgeries with the highest postoperative pain intensity for years [3], caesarean section surgery is often not given the importance and attention it deserves.

According to recent recommendations [12,21], the gold standard for intra- and postoperative analgesia after elective caesarean section surgery is co-adjuvant intrathecal morphine and systemic intravenous acetaminophen with NSAIDs. In this study, women receiving this regimen report postoperative pain rated at least moderate with movement at both 6 and 24 h. In fact, none of the five analgesic options analysed in this study were associated with average pain scores below 4/10 during the first 24 h with movement. This might indicate that we are still far from the ideal pain control in this population. Severe acute pain is a known risk factor for the development of chronic postsurgical pain, postpartum depression or increased consumption of opioids [7,8,22]. The authors believe that more optimal analgesic strategies should be developed to prevent these complications.

We conducted an open descriptive non-randomized non-controlled comparison of different anaesthetic and analgesic regimens, with the aim of identifying the most effective to provide the best pain relief. The women receiving intrathecal morphine (alone or in combination with fentanyl) reported superior pain relief than with fentanyl alone. The combination of intrathecal morphine and fentanyl along with IV conventional analgesics seemed to be the regimen associated with the lowest pain ratings, although this comparison did not reach statistical significance between all groups.

There is literature indicating that combining low doses of morphine (<150 μg) with a lipophilic opioid such as fentanyl or sufentanyl may be associated with lower pain scores and postoperative opioid consumption rates [23,24,25]. This combination of intrathecal opioids might present advantages such as (1) allowing a lower dose of intrathecal morphine, thus minimizing serious adverse effects such as respiratory depression, (2) improving early postoperative analgesia due to lipophilic opioids and (3) shortening the onset and improving the quality of the neuraxial blockade [25,26,27].

This study aimed to correlate pain scores with intrathecal morphine dosage. Some publications have shown that doses of morphine above 100 μg are not associated with better analgesic quality [28,29], while others demonstrated that it prolongs the time until the need for a first analgesic rescue [30]. This study does not corroborate the idea that doses beyond 100 µg of intrathecal morphine provide an additional benefit in immediate or delayed postoperative pain control with movement or at rest.

Higher intrathecal morphine doses might be associated with lesser yet clinically meaningful side effects such as pruritus [28] or nausea and vomiting [31]. There is scarce literature about the minimum recommended dose of intrathecal co-adjuvant morphine for post-caesarean analgesia. No differences in postoperative pain management were reported when 50, 100 or 150 micrograms of morphine were administered [28]. We believe that reports of morphine dosage should be correlated with BMI and not absolute doses. We found that women receiving doses of intrathecal morphine below 1 μg/kg had a statistically significant tendency towards higher postoperative pain ratings. The authors want to acknowledge that the failure to report opioid-related adverse events represents an important flaw of this research. Although most studies refer to doses between 50 and 100 mcg when referring to low doses of intrathecal morphine, there are no studies to the authors’ knowledge in which the dose of intrathecal morphine is assessed according to the anthropometric data of the patients. Therefore, further studies should be carried out to find the minimum effective analgesic dose to minimize the side effects of the drug.

The great interindividual variability in pain perception may explain why standardized treatments fail in some subgroups. Individualized analgesic regimens could improve postoperative outcomes [32]. These should be based on multimodal approaches including non-opioid analgesics, adjuvants and regional techniques which should be selected based on each patient’s condition. Importantly, individualization goes beyond drug and analgesic selection. It includes the assessment and titration of the analgesic regimen based on the patient response, functional objectives and his evolving clinical conditions. In fact, individualized analgesic regimens form part of the basis of the Enhanced-Recovery-After-Surgery protocols and are related with improved patient satisfaction, reduced complication rates, faster recovery and lower healthcare costs [33]. However, the identification of patients needing a more intense analgesic treatment may be a major challenge for the daily busy clinical practice.

Pain is a complex and subjective experience influenced by several factors including age, sex, genetic variability and psychological state. There is a growing interest in identifying the weight these variables carry in the development of severe postoperative pain. Factors such as younger age, presence of preoperative pain, female gender or a history of depression or anxiety symptoms have been associated with poorer postoperative pain control after various surgeries [34]. In women undergoing caesarean section, multiple studies have tried and failed to identify strong predictive variables [35]. In this study, the only variable we found to be independently associated with early postoperative pain was alcohol consumption, but the authors think that it could be a spurious correlation.

This study bears some limitations. The patients’ pain at 4 and 12 h after surgery was not assessed, which could have provided more information about the impact of the different analgesic options on early postoperative pain scores and how they evolved during the following hours. Similarly, pain at 48 h postoperatively was not assessed, which limits the ability to evaluate the efficacy of certain analgesic combinations independently of the residual effects of intrathecal morphine or fentanyl. Likewise, the total requirements and frequency of analgesic rescue during the first 24 h after caesarean section have not been recorded in all the hospitals, therefore they were not analysed. Some of the analgesic groups presented had a low number of patients. It is therefore plausible that a larger sample size within certain subgroups could reveal more pronounced differences between groups.

For instance, the combination of fentanyl and intrathecal morphine with acetaminophen and NSAIDs appears to consistently provide superior analgesic efficacy compared to other therapeutic options, apart from its comparison to fentanyl alone at 24 h during mobilization. Nonetheless, statistically significant differences were observed in only four of the comparisons conducted. Moreover, for the same reason, there is a possibility that some of the observed differences may not be truly statistically significant, but rather attributable to random variation or insufficient statistical power. It is also worth acknowledging that other analgesic strategies, such as regional techniques including the TAP block or the quadratus lumborum block, were not evaluated due to an insufficient sample size. This likely introduced variability in the treatment effect estimates between analgesic strategies.

In conclusion, the results indicate that there is currently no optimal management for acute postoperative pain following caesarean sections, underscoring the need for the continued development of new analgesic strategies. Although these results do not allow us to make a categorical statement, among the different analgesic options the authors recommend the use of the intrathecal combination of morphine together with fentanyl, without exceeding a 100 μg dose of morphine but above 1 μg/kg. However, it is necessary to develop RCT to assess whether this is really the best analgesic option. No risk factor associated with poorer outcomes was found, although further studies are needed to individualize the therapeutic approach in the future.

## Figures and Tables

**Table 1:** Population sample descriptive analysis (n = 508).

Variables		
Weight (kg)		80.63 (15.93)
Height (cm)		163.75 (27.64)
Age (years)		35.98 (5.52)
BMI		30.5 (6.14)
Alcohol consumption	Yes	22 (4.33)
	No	486 (95.67)
Smoker	Yes	58 (11.42)
	No	371 (73.03)
	Ex	79 (15.55)
Drug consumption	Yes	7 (1.38)
	No	490 (96.45)
	Ex	11 (2.17)
ASA	I	293 (57.68)
	II	201 (39.57)
	III	14 (2.75)
	IV	0 (0.0)
Obstetric risk ^a^	No	177 (34.84)
	Gestational diabetes	69 (13.58)
	Hypertensive states	36 (7.09)
	Multiparity	121 (23.82)
	Alterations in coagulation	9 (1.77)
	Placenta issues	33 (6.5)
	Multiple foetus	30 (5.91)
	Foetal dystocias	44 (8.66)
	Other	74 (14.57)
Pain in previous operations	Yes	119 (23.43)
	No	389 (76.57)
Previous caesarean	Yes	261 (51.38)
	No	247 (48.62)
Stillbirth	Yes	88 (17.32)
	No	420 (82.68)

Values presented as mean (standard deviation), median (IQR) or frequency (%). BMI, Body Mass Index; ASA, American Society of Anaesthesiology. ^a^ The obstetric risk percentage is relative to the total number of pregnancies.

**Table 2:** Sample description and test results on postoperative pain according to intrathecal technique.

Variable	Intrathecal Technique	n ^a^	VNRS	Fentanyl		Morphine		Morphine + Fentanyl	
				*p*-Value	Effect Size	*p*-Value	Effect Size	*p*-Value	Effect Size
Pain after 6 h at rest	Fentanyl	152	3.59 (2.7)	-	-	>0.001	−0.435	0.012	−0.088
	Morphine	152	2.44 (2.6)	>0.001	0.435	-	-	0.512	0.382
	Morphine + fentanyl	176	2.65 (2.24)	0.012	0.088	0.512	−0.382	-	-
Pain after 6 h with movement	Fentanyl	152	5.71 (2.71)	-	-	0.004	−0.370	0.004	−0.044
	Morphine	152	4.66 (2.97)	0.004	0.370	-	-	1.000	0.360
	Morphine + fentanyl	176	4.78 (2.48)	0.004	0.044	1.000	−0.360	-	-
Pain after 24 h at rest	Fentanyl	152	2.64 (2.26)	-	-	1.000	−0.011	1.000	−0.008
	Morphine	152	2.61 (2.55)	1.000	0.011	-	-	1.000	0.003
	Morphine + fentanyl	176	2.63 (2.20)	1.000	0.008	1.000	−0.003	-	-
Pain after 24 h with movement	Fentanyl	152	5.07 (2.43)	-	-	0.938	0.109	1.000	0.067
	Morphine	152	5.34 (2.55)	0.938	−0.109	-	-	1.000	−0.047
	Morphine + fentanyl	176	5.18 (2.28)	1.000	−0.067	1.000	0.047	-	-

Values presented as mean (standard deviation), median. VNRS, Verbal Numerical Rate Scale. ^a^ Total number of patients.

**Table 3:** Sample description and test results of postoperative pain according to intrathecal technique and postoperative analgesia.

Variable	Technique	n ^a^	VNRS	Intrathecal Fentanyl + Acetaminophen + NSAIDs (IF)		Intrathecal Fentanyl + Elastomeric Pump (IF + EL)		Intrathecal Fentanyl + Epidural Analgesia (IF + EP)		Intrathecal Morphine + Acetaminophen + NSAIDs (IM)		Intrathecal Fentanyl and Morphine + Acetaminophen + NSAIDs (IF + IM)	
				*p*-Value	Effect Size	*p*-Value	Effect Size	*p*-Value	Effect Size	*p*-Value	Effect Size	*p*-Value	Effect Size
Pain after 6 h at rest	IF	74	3.91 (2.75)	-	-	1.00	−0.072	0.45	0.394	0.06	0.390	0.01	0.610
	IF + EL	29	4.10 (2.86)	1.00	0.072	-	-	0.36	0.478	0.12	0.464	0.04	0.733
	IF + EP	49	2.88 (2.44)	0.45	−0.394	0.36	−0.478	-	-	1.00	0.006	1.00	0.180
	IM	116	2.86 (2.65)	0.06	−0.390	0.12	−0.464	1.00	−0.006	-	-	1.00	0.158
	IF + IM	124	2.49 (2.04)	0.01	−0.610	0.04	−0.733	1.00	−0.180	1.00	−0.158	-	-
Pain after 6 h with movement	IF	74	5.49 (2.62)	-	-	1.00	−0.239	1.00	−0.065	1.00	0.087	0.06	0.421
	IF + EL	29	6.14 (3.08)	1.00	0.239	-	-	1.00	0.178	0.70	0.314	0.01	0.671
	IF + EP	49	5.65 (2.57)	1.00	0.065	1.00	−0.178	-	-	1.00	0.149	0.02	0.496
	IM	116	5.25 (2.79)	1.00	−0.087	0.70	−0.314	1.00	−0.149	-	-	0.09	0.311
	IF + IM	124	4.44 (2.41)	0.06	−0.421	0.01	−0.671	0.02	−0.496	0.09	−0.311	-	-
Pain after 24 h at rest	IF	74	2.77 (2.23)	-	-	1.00	−0.025	1.00	0.089	1.00	−0.038	1.00	0.150
	IF + EL	29	2.83 (2.49)	1.00	0.025	-	-	1.00	0.109	1.00	−0.014	1.00	0.176
	IF + EP	49	2.57 (2.30)	1.00	−0.089	1.00	−0.109	-	-	1.00	−0.118	1.00	0.056
	IM	116	2.86 (2.55)	1.00	0.038	1.00	0.014	1.00	0.118	-	-	1.00	0.178
	IF + IM	124	2.45 (2.07)	1.00	−0.150	1.00	−0.176	1.00	−0.056	1.00	−0.178	-	-
Pain after 24 h with movement	IF	74	4.92 (2.28)	-	-	1.00	−0.189	1.00	−0.160	0.33	−0.308	1.00	−0.067
	IF + EL	29	5.38 (2.88)	1.00	0.189	-	-	1.00	0.037	1.00	−0.107	1.00	0.128
	IF + EP	49	5.29 (2.35)	1.00	0.160	1.00	−0.037	-	-	1.00	−0.151	1.00	0.093
	IM	116	5.65 (2.43)	0.33	0.308	1.00	0.107	1.00	0.151	-	-	0.28	0.244
	IF + IM	124	5.07 (2.30)	1.00	0.067	1.00	−0.093	1.00	−0.128	0.28	−0.244	-	-

Values presented as mean (standard deviation), median. VNRS, Verbal Numerical Rate Scale. ^a^ Total number of patients.

**Table 4:** Results on postoperative pain and intrathecal morphine dosage.

Variable	Morphine Dose	n ^a^	Mean ^b^	*p* Value	Effect Size
Pain after 6 h at rest	dose > 100 mcg	19	2.11 (2.60)	0.52	0.148
	dose ≤ 100 mcg	133	2.49 (2.61)		
	dose > 1 mcg/kg	115	2.27 (2.70)	0.023	0.287
	dose ≤ 1 mcg/kg	37	3.00 (2.21)		
Pain after 6 h with movement	dose > 100 mcg	19	4.74 (3.11)	0.96	0.028
	dose ≤ 100 mcg	133	4.65 (2.96)		
	dose > 1 mcg/kg	115	4.42 (3.07)	0.07	0.346
	dose ≤ 1 mcg/kg	37	5.43 (2.52)		
Pain after 24 h at rest	dose > 100 mcg	19	2.68 (2.54)	0.88	0.033
	dose ≤ 100 mcg	133	2.60 (2.56)		
	dose > 1 mcg/kg	115	2.49 (2.49)	0.07	0.203
	dose ≤ 1 mcg/kg	37	3.00 (2.79)		
Pain after 24 h with movement	dose > 100 mcg	19	5.37 (2.69)	0.80	0.012
	dose ≤ 100 mcg	133	5.34 (2.53)		
	dose > 1 mcg/kg	115	5.17 (2.57)	0.16	0.274
	dose ≤ 1 mcg/kg	37	5.86 (2.43)		

Values presented as mean (standard deviation), median. ^a^ Total number of patients. ^b^ Pain expressed as 0–10 on Verbal Numerical Rate Scale.

## Data Availability

De-identified data may be requested with reasonable justification from the authors (by email to the corresponding author) and will be shared after approval as per the authors’ Institution policy.
